# Graphene Oxide–Polyphenylsulfone Nanocomposite Beads for Paracetamol Removal from Aqueous Solution

**DOI:** 10.3390/membranes14010009

**Published:** 2023-12-28

**Authors:** Mansour Alhoshan, Arun Kumar Shukla, Javed Alam, Ali Awadh Hamid

**Affiliations:** 1 Department of Chemical Engineering, College of Engineering, King Saud University, Riyadh 11451, Saudi Arabia; mhoshan@ksu.edu.sa (M.A.);; 2 King Abdullah Institute for Nanotechnology, King Saud University, Riyadh 11451, Saudi Arabia; ashukla@ksu.edu.sa

**Keywords:** polymeric beads, nanocomposite, graphene oxide, paracetamol removal, pharmaceutical contaminants

## Abstract

This study introduces a promising and practical method for the removal of paracetamol from aqueous environments, employing graphene oxide–polymer nanocomposite beads. The approach involves the utilization of a straightforward and facile phase inversion method, offering a convenient and efficient one-step process for the creation of adsorbent beads by integrating polymers and graphene oxide (GO). The synthesized nanocomposite beads are tailored for the removal of paracetamol from simulated wastewater in batch systems. Extensive characterization techniques including XPS, FTIR, SEM, TGA, and zeta potential analysis are employed to scrutinize the chemical properties and structural attributes of the prepared beads. The investigation explores the impact of critical parameters such as adsorbent dosage, adsorption duration, initial paracetamol concentration, and solution pH on the adsorption process. These nanocomposite beads exhibit an exceptional paracetamol removal efficiency, achieving up to 99% removal. This research not only contributes to the advancement of efficient and sustainable adsorbent materials for pollutant removal but also underscores their potential for environmentally friendly and cost-effective solutions in the domain of wastewater treatment.

## 1. Introduction

Pharmaceutical drugs have long been hailed as essential components of modern healthcare, serving a pivotal role in the treatment of various illnesses in both human and animal populations [1]. These drugs are ingrained in our daily lives, offering relief from pain, fever, and a multitude of ailments [2]. However, the widespread use of pharmaceuticals has inadvertently led to a concerning environmental issue—the continuous discharge of these compounds into our water bodies [3,4]. Hospitals, households, and pharmaceutical production plants contribute to this steady stream of pharmaceutical contaminants entering our water resources. This phenomenon has transformed pharmaceuticals into emerging contaminants, raising alarm bells due to their potential to generate adverse effects, which could be detrimental to both human health and aquatic ecosystems [3,5,6,7,8,9,10].

Among the pharmaceutical compounds infiltrating aquatic environments, paracetamol stands out as one of the most frequently detected substances. As a widely used analgesic and antipyretic drug, it has earned a ubiquitous presence across the globe for its ability to alleviate pain and reduce fever [11,12]. While the presence of paracetamol in water and wastewater has been detected in relatively low concentrations, it is worth noting that much higher concentrations have been measured in waste effluents worldwide [13]. This pervasive presence of paracetamol in aquatic systems underscores the urgency of developing efficient methods for its removal [10,14,15,16].

Addressing the growing concern over pharmaceutical contaminants in water, various removal techniques have been devised and implemented to combat this ecological challenge. These methods encompass bioreactors, degradation and photodegradation, filtration, photocatalysis, photo-Fenton, Fenton-like reactions, coagulation, and adsorption [15,17,18,19,20,21]. Among these approaches, adsorption has emerged as a widely embraced and highly efficient method for wastewater treatment [11,14,17,22]. Its appeal lies in its affordability, ease of operation, remarkable efficiency, high capacity, simplicity, reliability, and minimal energy consumption.

In the realm of adsorption, a diverse array of adsorbent materials has been explored for the removal of pollutants from water. Activated carbon, charcoal, plant derivatives, fly ash, metal nanoparticles, and chitosan are just a few examples of the materials that have found application in this context [23,24,25,26,27]. However, one material has been attracting significant attention in recent years for its remarkable properties—graphene oxide (GO). GO boasts an extensive surface area, accessible surface sites, high hydrophobicity, and impressive mechanical characteristics, making it a promising candidate for adsorption applications. Researchers have explored the potential of graphene-based materials for adsorbing various pollutants, capitalizing on its delocalized π–π electron system, which forms strong bonds with a wide range of contaminants [2,15,28,29,30].

However, even with these promising attributes, many of the materials, including GO, have inherent limitations. These limitations encompass low adsorption capacity, higher costs, frequent regeneration requirements, and the propensity to leach into the treated water, necessitating additional post-treatment for separation [31,32,33]. Moreover, these materials often consist of nano-sized, uneven particles, which can lead to operational issues in adsorption columns, such as channeling, bed clogging, elevated pressure drops, reduced performance, and increased operational costs [34]. Consequently, there arises a need for the development of a suitable adsorbent material that can be seamlessly integrated into fixed bed continuous adsorption columns, addressing these concerns [35].

To tackle these challenges and facilitate the recycling of GO particles, a promising avenue is to composite them with polymers [36]. Among these polymers, polyphenylsulfone (PPSU) has garnered considerable attention for its compatibility with GO and its potential to enhance the overall adsorption efficiency. Various techniques, such as high internal phase emulsions, have been employed to produce beads and spherical nanocomposites [35,37]. However, these methods often involve high-temperature processes, protracted and intricate procedures, which can hinder their practicality.

In this study, we present a straightforward and facile approach to synthesize nanocomposite beads using a phase inversion method, which offers an expedient, uncomplicated, and single-step method for creating adsorbent beads through the integration of polymers and GO. The prepared nanocomposite beads are designed for the removal of paracetamol from simulated wastewater in batch systems. We will explore the chemical properties and structural characteristics of these materials through various characterization techniques. Additionally, the study will delve into the impact of different parameters, including adsorbent dosage, initial paracetamol concentration, and solution pH, on the adsorption process in batch systems. By addressing the limitations of current adsorbents and offering a cost-effective, eco-friendly alternative, this work contributes to the broader efforts to safeguard our water resources and protect the health of both humans and aquatic ecosystems.

## 2. Materials and Methods

### 2.1. Materials

The materials used in this research encompassed polyphenylsulfone (PPSU, Ultrason P 3010) as the polymer, generously supplied by BASF (Berlin, Germany), while graphene oxide (GO) and the pharmaceutical component paracetamol were procured from Sigma Aldrich (Kenilworth, NJ, USA). The procured nanostructure GO specification included functional groups such as carboxyl, hydroxyl or epoxy, and C/O-2.3. To facilitate the formulation process, polyethylene glycols (PEGs 6000) and N-methyl-2-pyrrolidone (NMP) were sourced from Merck in Darmstadt, Germany. The essential physicochemical properties of the paracetamol compound are succinctly summarized in Table 1, providing insight into key attributes such as molecular weight and solubility. Throughout the duration of the investigation, deionized (DI) water was consistently obtained from a Milli-Q system (Millipore, Burlington, MA, USA), ensuring the requisite water purity for the experiments.

### 2.2. Preparation of Polymeric Nanocomposite Beads

**Figure 1:**
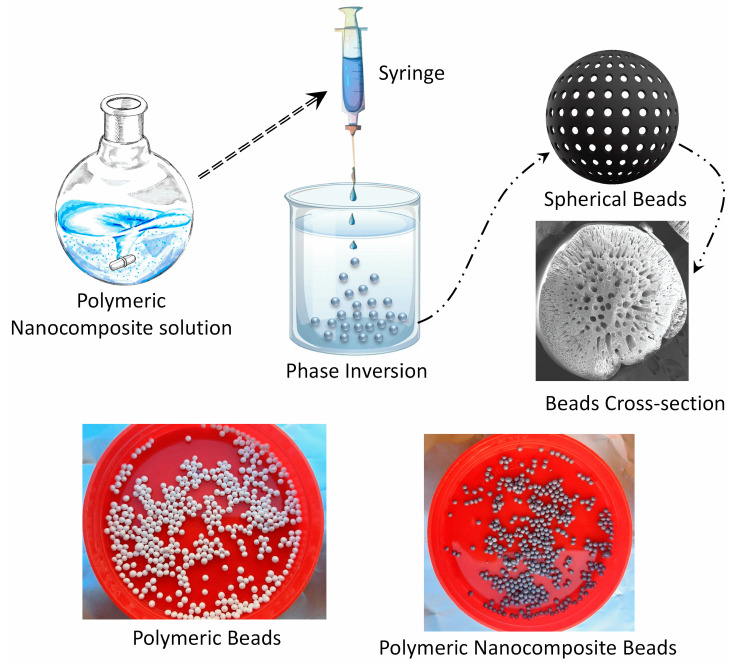
Schematic diagram illustrating the step-by-step process involved in the preparation of polymeric nanocomposite beads.

The synthesis of polymeric nanocomposite beads of PPSU/GO was achieved through a well-defined phase inversion approach, as outlined in this research study. Initially, a polymeric solution was meticulously prepared by blending 15 wt% PPSU and 3 wt% g PEG within an 82 wt% NMP solvent with the entire mixture undergoing thorough mixing at a controlled temperature of 60 °C for an extended period of 24 h. Subsequently, to facilitate the formation of these nanocomposite beads, the distance between the water surface and the syringe tip, which had a diameter of 0.5 mm, was precisely set at 4 cm. The prepared polymeric solution was then gently and vertically pumped into a vessel of deionized water, employing a slow, drop-by-drop technique. This controlled introduction of the polymeric solution into the deionized water initiated a solvent-to-nonsolvent exchange, prompting the immediate generation of polymeric spherical beads, each measuring approximately 2 mm in diameter. Following this stage, these newly formed beads were allowed to remain submerged in water for an additional 24 h to ensure complete solvent removal. Subsequently, the beads were carefully collected and dried at room temperature. In the case of the polymeric nanocomposite beads of PPSU/GO, the same procedure was employed, but with a modification: 1 wt% of GO particles, maintaining an equal weight ratio, were uniformly dispersed within the polymeric solution. Once again, the distance between the water surface and the syringe tip was set at 4 cm, and the polymeric solution containing the integrated GO particles was introduced into the deionized water drop by drop. The culmination of this process led to the formation of nanocomposite beads with GO incorporation, which were subsequently dried at room temperature. A schematic diagram and photographic images of the preparation of beads are presented in Figure 1.

### 2.3. Characterization of Adsorbents Beads

In the comprehensive characterization of the adsorbent beads employed in this research, a series of sophisticated analytical techniques were employed to unveil their structural and surface properties. Surface elemental compositions and chemical bonding on the prepared polymeric beads were quantified using X-ray photoelectron spectroscopy (XPS), which was carried out with a JEOL spectrometer (JEOL, JPS-9030, Tokyo, Japan). This XPS analysis utilized a Mg Kα (700 eV) X-ray source, operated at 10 mA and 12 kV, within an ultra-high vacuum environment (<10^−7^ Pa). Furthermore, the structural characteristics were explored through Fourier-transform infrared spectroscopy (FTIR) within a wavenumber range of 600–4000 cm^−1^ using a Vertex 80 instrument by Bruker in the UK. To assess the physical morphology of the prepared beads, scanning electron microscopy (SEM) was employed, utilizing a JEOL instrument from Tokyo, Japan. Additionally, the thermal and oxidative stability of these beads were evaluated using a thermogravimetric analyzer (TGA) from Mettler Toledo, Vienna, Austria, where samples of the prepared beads were analyzed under a nitrogen atmosphere with a constant flow rate of 40 mL/min and a heating rate of 20 °C/min, ranging from 100 to 700 °C. The charging properties, including zeta potential and isoelectric point, were determined using a SurPASS electrokinetic analyzer by Anton Paar in Graz, Austria, measuring tangential streaming potential. The zeta potential measurements were conducted in a 1 mM KCl electrolyte solution across a range of pH values. The pH was adjusted using 0.05 M HCl and 0.05 M NaOH solutions, and the resulting zeta potential values were calculated using the Attract^®^ 2.0 software based on the streaming potential measurements, applying the Fairbrother–Mastin relationship. This thorough characterization process provided a detailed understanding of the adsorbent beads’ physical, chemical, and electrokinetic properties, which is crucial for their application in the subsequent adsorption studies.

### 2.4. Batch Paracetamol Adsorption Studies

In the batch adsorption studies, the synthetic wastewater used in this research was meticulously prepared by dissolving 1000 mg of paracetamol in 1000 mL of deionized water, resulting in various paracetamol concentrations. The adsorption behavior of paracetamol was investigated using polymeric nanocomposite beads of PPSU/GO and pure PPSU as adsorbents. To facilitate the adsorption process, the adsorbent beads were brought into contact with the paracetamol-containing solutions in a thermostatic shaker, which operated at a constant speed of 150 rpm. The optimal conditions were determined by varying the adsorbent dosages within the range of 0.1–0.5 g, employing paracetamol concentrations spanning 25–200 mg/L, and adjusting the pH values over a wide range from 4 to 9, all conducted at room temperature. Upon reaching equilibrium at the conclusion of the adsorption process, samples were extracted at different time intervals. The remaining concentrations of paracetamol in the solutions were assessed through quantification using a UV-Vis spectrophotometer (Cary 60 UV-Vis, Agilent Technologies, Santa Clara, CA, USA). This measurement was conducted at the optimal wavelengths for paracetamol, precisely at 243 nm, following the determination of absorbance wavelengths for paracetamol across the spectrum from 200 to 400 nm. The experiments were conducted twice, and the average values from these trials were recorded as data points. The quantity of adsorbed paracetamol at equilibrium and the adsorption efficiency were subsequently calculated using the relevant equations.

$$Equilibrium\;point=\frac{\left(C_{i\;}-C_{f}\right)\times V}{m}$$

$$Adsorption\;efficiency\;(\%)=\frac{C_{i\;}-C_{f}}{C_{i}}\times 100$$

## 3. Results and Discussion

### 3.1. Adsorbent Bead Characterization

The synthesized polymeric nanocomposite beads, with the inclusion of graphene oxide (GO), displayed distinct physicochemical properties in comparison to their counterparts lacking GO. This variation strongly implies the successful incorporation of GO within the polymeric matrix. To verify this, several specialized chemical analysis techniques, including X-ray photoelectron spectroscopy (XPS) and Fourier-transform infrared spectroscopy (FTIR), were employed to examine and characterize the chemical composition of both the polymeric and nanocomposite beads. Furthermore, additional surface morphology and charge and thermal analyses were conducted using scanning electron microscopy (SEM), zeta potential and thermogravimetric analysis (TGA), respectively. These comprehensive characterizations offer valuable insights into the material properties and chemical functionalities of the adsorbent beads, shedding light on the impact of GO incorporation on their physicochemical attributes.

#### 3.1.1. Spectral Analysis

**Figure 2:**
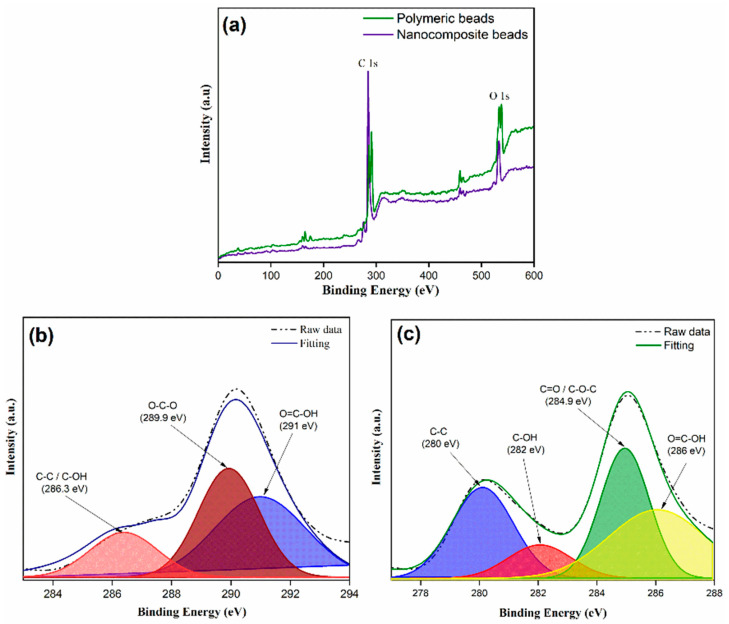
XPS spectra illustrating a comparative analysis between polymeric and nanocomposite beads; (**a**) XPS survey, (**b**) C1s core level spectra for polymeric beads, (**c**) C1s core level spectra for nanocomposite beads.

XPS analysis provided valuable insights into the chemical composition of the polymeric beads, shedding light on their surface elemental composition and chemical bonding. The XPS spectra depicted in Figure 2a reveal prominent elemental peaks on the beads’ surface, with oxygen (O 1s) and carbon (C 1s) peaks discerned at binding energies of 535 eV and 287 eV, respectively. To gain a deeper insight into the chemical moieties present, high-resolution C 1s core level XPS spectra of both the polymeric and nanocomposite beads were analyzed, as illustrated in Figure 2b,c). The fitting of these spectra involved a combination of Gaussian and Lorentzian functions, allowing for a more detailed deconvolution of the carbon peaks. Notably, with the incorporation of graphene oxide (GO) into the polymer matrix, the C 1s spectrum exhibited two distinctive carbon peaks. Specifically, the binding energies at 280 eV, 282 eV, 284.9 eV, and 286 eV were attributed to C-H, C-OH, C=O/C-O-C, and O=C-OH functionalities, respectively. The emergence of these unique carbon peaks is indicative of the successful integration of GO within the polymeric matrix. This alteration in surface chemistry is of utmost significance, as it influences the adsorption behavior of the beads. The presence of oxygen-containing groups on the bead’s surface, such as C-OH, C=O, and O=C-OH, suggests enhanced reactivity toward adsorbates like paracetamol. These functional groups can participate in hydrogen bonding and electrostatic interactions, contributing to the enhanced adsorption efficiency and selectivity of the adsorbent material. The presence of C-H groups, on the other hand, may contribute to hydrophobic interactions with organic molecules. Thus, the detailed XPS analysis lays the foundation for understanding the chemical mechanisms and surface functionalities that underpin the adsorption process, setting the stage for the subsequent discussions on adsorption performance and behavior.

**Figure 3:**
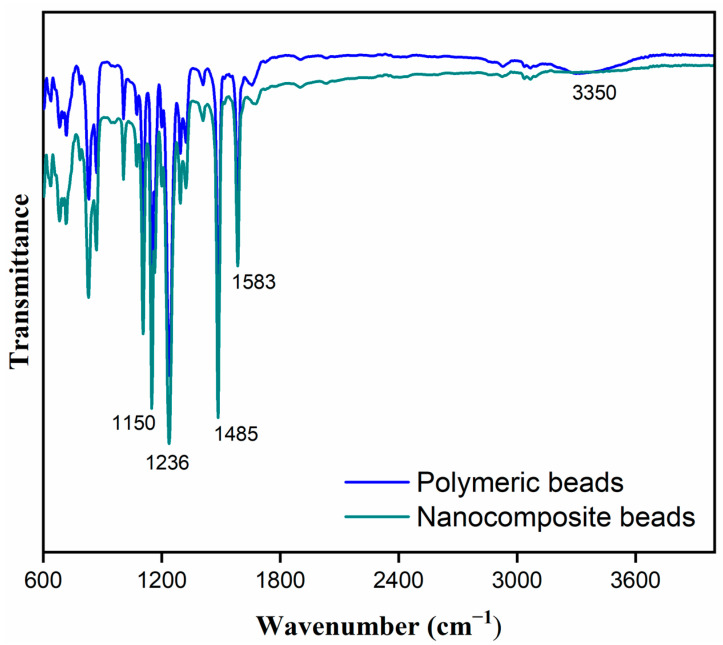
Fourier transform infrared (FTIR) spectra illustrating a comparative analysis between polymeric and nanocomposite beads, highlighting distinctive spectral features and differences in chemical composition.

The FTIR analysis provided further insights into the structural and chemical characteristics of both the polymeric and nanocomposite beads. As depicted in Figure 3, the FTIR spectra revealed several distinct peaks, each corresponding to specific functional groups. Notably, prominent peaks were observed at 1150 cm^−^¹, indicative of C-O stretching, and at 1150 cm^−^¹, representing C=O stretching. These peaks are associated with the presence of carbonyl (C=O) and ether (C-O) groups, which are characteristic of the chemical composition of the beads. Additionally, the appearance of peaks at 1236 cm^−^¹ (C-O) and 1485 cm^−^¹ (C-C) further supports the presence of oxygen-containing functional groups in the material. The peaks at 1583 cm^−^¹ (C=C) signify the presence of carbon–carbon double bonds, which could be attributed to the carbon framework of the polymer and graphene oxide components. Moreover, the broad peak at 3350 cm^−^¹ (O-H) is indicative of hydroxyl groups, highlighting the presence of surface hydrophilicity. The presence of C=O, C-O, and O-H functional groups suggests a high potential for hydrogen bonding and other interactions with polar adsorbates, such as paracetamol, which contains hydroxyl and carbonyl groups. These interactions can significantly influence the adsorption capacity and selectivity of the beads as well as their affinity for specific pollutants. Additionally, the presence of carbon–carbon double bonds (C=C) indicates the carbon-rich nature of the material, which is crucial for adsorption processes. The FTIR analysis provides critical information about the surface chemistry and functionality of the beads, which directly correlates with their adsorption behavior and performance.

#### 3.1.2. Morphological Characteristics of Beads

**Figure 4:**
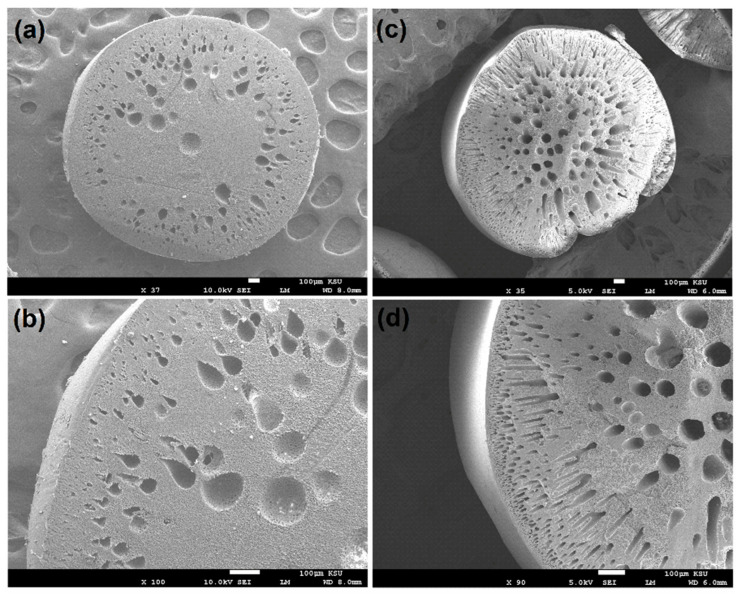
Cross-section morphology depicting: (**a**,**b**) polymeric beads and (**c**,**d**) nanocomposite beads, showcasing the internal structure and characteristics of both materials at different magnifications.

The SEM images in Figure 4 provided a detailed view of the morphological characteristics of both the polymeric and nanocomposite beads. These images revealed significant alterations in the bead structures following the incorporation of GO into the polymeric matrix. In particular, Figure 4c,d distinctly illustrated the pronounced changes in the morphology of the nanocomposite beads compared to the polymeric counterparts. The images demonstrated that the nanocomposite beads exhibited a spherical structure with a cross-sectional morphology characterized by interconnected macroscale grooves. In contrast, the polymeric beads displayed a more conventional cross-sectional structure. These morphological changes are indicative of the profound impact of GO incorporation on the physical structure of the beads. The formation of interconnected macroscale grooves in the nanocomposite beads suggests a more intricate and porous surface architecture, which can be highly advantageous for adsorption applications. These microstructural changes potentially enhance the accessible surface area and provide more sites for interactions with adsorbate molecules, thereby contributing to the improved adsorption performance observed in the experiments. Furthermore, the structural characteristics of nanocomposite beads were demonstrated to exhibit notable mechanical stability, including toughness, as evidenced by the conducted mechanical compression tests. The structural modifications depicted in the SEM images are a testament to the role of nanocomposite design in tailoring the physical properties of adsorbent materials for enhanced efficiency in wastewater treatment and other environmental remediation processes.

#### 3.1.3. Surface Charge (Zeta Potential) Analysis

**Figure 5:**
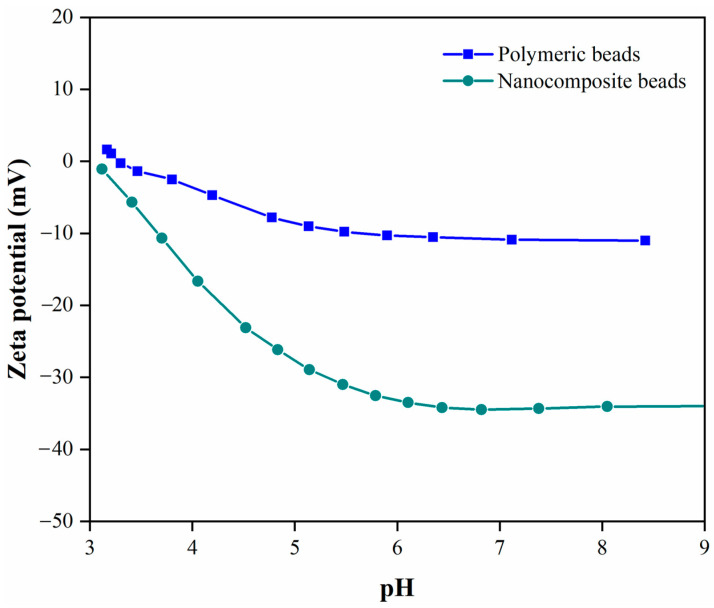
Comparative analysis of surface charge properties between polymeric beads and nanocomposite beads, highlighting differences in their surface charge characteristics.

The zeta potential analysis is crucial in understanding the surface charge properties of the polymeric and nanocomposite beads, shedding light on their electrokinetic characteristics. By measuring the streaming potential using electrolyte solutions at different pH levels, we gained insights into the behavior of these materials in aqueous environments. As depicted in Figure 5, it is evident that the zeta potential of both the polymeric and nanocomposite beads exhibited a negative charge, which became more pronounced with increasing pH. This trend is indicative of the dominance of negatively charged functional groups on the bead surfaces. Notably, the incorporation of GO into the polymer matrix had a discernible effect on the zeta potential. At pH 7, the zeta potential of the polymeric beads was measured at −10 mV, while the nanocomposite beads exhibited a considerably enhanced zeta potential of −34 mV. This increase in negative zeta potential suggests that the incorporation of GO introduced additional functional groups that enhance the surface charge density of the nanocomposite beads. This observation holds significant implications for the adsorption behavior, as a higher zeta potential can lead to increased electrostatic interactions between the adsorbent and charged species in the wastewater. Therefore, the zeta potential results underscore the importance of the nanocomposite’s surface charge characteristics in influencing its paracetamol adsorption performance.

#### 3.1.4. Thermogravimetric Analysis

**Figure 6:**
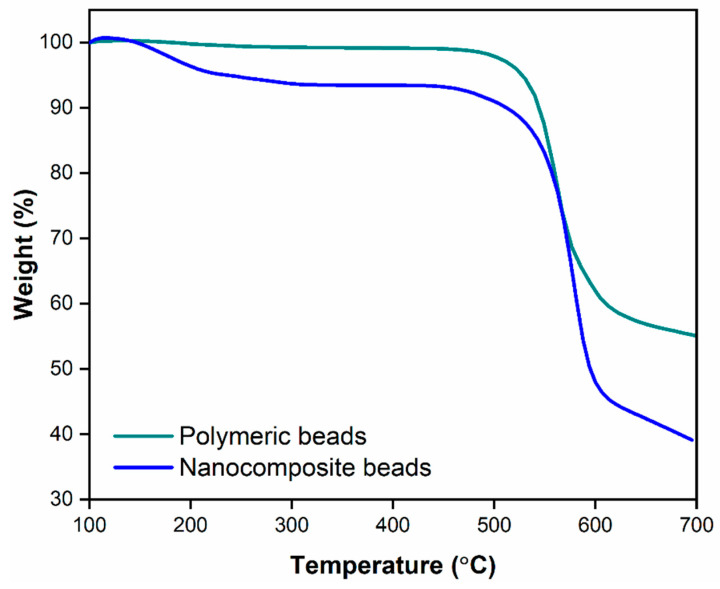
Thermogravimetric analysis illustrating the comparative thermal properties of polymeric beads and nanocomposite beads, showcasing their respective thermal stability and decomposition profiles.

The TGA results, as illustrated in Figure 6, provided valuable insights into the thermal stability and decomposition behavior of the polymeric and nanocomposite beads. It is evident that the nanocomposite beads exhibited a notably higher level of thermal stability and demonstrated lower weight loss compared to the polymeric beads when subjected to a temperature range of 100 to 700 °C. This improved thermal stability is attributed to the presence of GO within the polymeric matrix. The interaction between GO and the polymer matrix is notably strong, resulting in enhanced thermal properties in the nanocomposite beads. This interaction is likely due to the formation of covalent and non-covalent bonds between GO and the polymer, which reinforce the structural integrity and reduce the susceptibility to thermal decomposition. Consequently, the incorporation of GO within the polymeric matrix contributes to the overall enhancement of the nanocomposite beads’ thermal stability. This aspect is of significant importance, as it ensures the material’s durability and effectiveness when used in practical applications, such as wastewater treatment, where it may be exposed to varying temperatures and conditions. The TGA results thus confirm the positive influence of GO on the thermal properties of the nanocomposite beads, which will be further discussed in terms of its implications on adsorption performance and reusability.

### 3.2. Batch Adsorption Studies

The study’s focus on developing an efficient adsorbent for the removal of pollutants, such as paracetamol, is crucial in addressing environmental contamination issues. While pure GO nanoparticles have shown effective pollutant removal capabilities, their application in powder form is often hindered by challenges related to dispersion and separation from liquid media due to their hydrophobic nature. This limitation necessitates the use of additional equipment, like centrifuges, for efficient separation. To overcome these issues, the incorporation of GO into a polymeric matrix was explored. In this regard, the polymeric and nanocomposite beads were subjected to batch adsorption studies to evaluate their potential for paracetamol removal from wastewater.

#### 3.2.1. Effect of Adsorbent Dosage

The investigation into the effect of adsorbent dosage is a critical aspect of understanding the adsorption process’s efficiency and optimizing the use of polymeric and nanocomposite beads for paracetamol removal from wastewater. In this study, varying amounts of both types of adsorbents were introduced into 25 mL of simulated paracetamol-containing wastewater, initially at a concentration of 100 mg/L. These mixtures were stirred at ambient temperature and a pH of 7 to assess the impact of adsorbent dosage on paracetamol removal.

**Figure 7:**
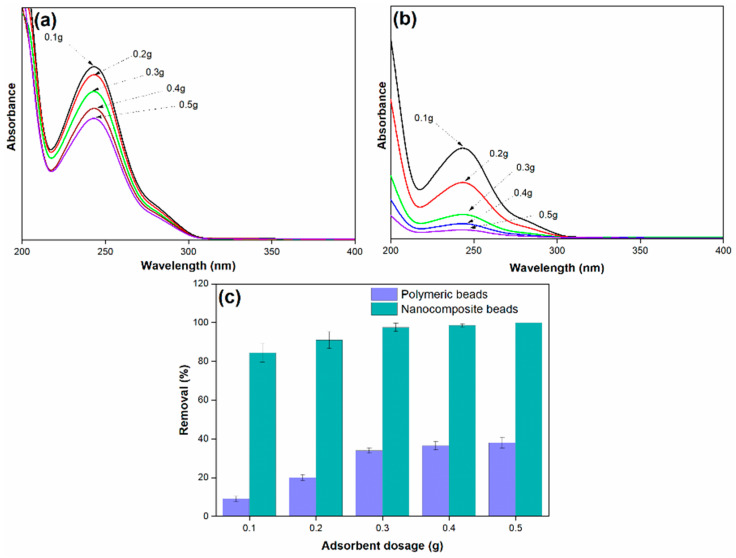
(**a**) Adsorption spectra of paracetamol on polymeric beads at varying adsorbent dosages, (**b**) adsorption spectra of paracetamol on nanocomposite beads at varying adsorbent dosages, and (**c**) paracetamol removal efficiency at varying adsorbent dosages (adsorbent dosage = 0.1 g to 0.5 g, concentration = 100 mg/L, pH = 7, solution volume = 25 mL).

The results, as depicted in Figure 7, clearly demonstrate that the efficiency of paracetamol removal increases as the mass of the adsorbent is augmented. This relationship is fundamentally associated with the concept of adsorptive surface area. An increased dosage of adsorbent provides a larger surface area available for interaction with paracetamol molecules, offering a greater number of active surface sites for adsorption. Consequently, the removal of paracetamol is enhanced as a direct result of this increased surface area. It is noteworthy that the highest amount of paracetamol removal, approximately 99%, was achieved when using 0.5 g of adsorbent dosage. However, it is equally important to observe that the use of 0.3 g of nanocomposite beads yielded a comparable result and showed approximately 98%. This finding underscores the efficiency of the nanocomposite beads, as a slightly lower dosage still provided similar removal capabilities. This observation has practical significance, as it suggests that a lower amount of nanocomposite adsorbent can achieve the same removal efficiency as a larger quantity of polymeric beads. Therefore, 0.3 g of the nanocomposite beads was deemed sufficient and was chosen as the optimum adsorbent dosage for further investigations.

The mechanism underlying this phenomenon is grounded in the fundamental principles of adsorption. As the dosage of adsorbent increases, the available surface area for interaction with the adsorbate (paracetamol) expands, resulting in a higher number of active binding sites. These binding sites, characterized by the surface functionalities identified in the earlier characterizations (e.g., oxygen-containing groups), enable the adsorbent to effectively attract and capture paracetamol molecules. The electrostatic interactions and other attractive forces between the adsorbent surface and paracetamol contribute to the enhanced removal efficiency. The versatility and effectiveness of the nanocomposite beads are particularly noteworthy, as they provide comparable performance with a smaller quantity, suggesting their potential for cost-effective and environmentally friendly pollutant removal applications.

#### 3.2.2. Effect of Adsorption Time

**Figure 8:**
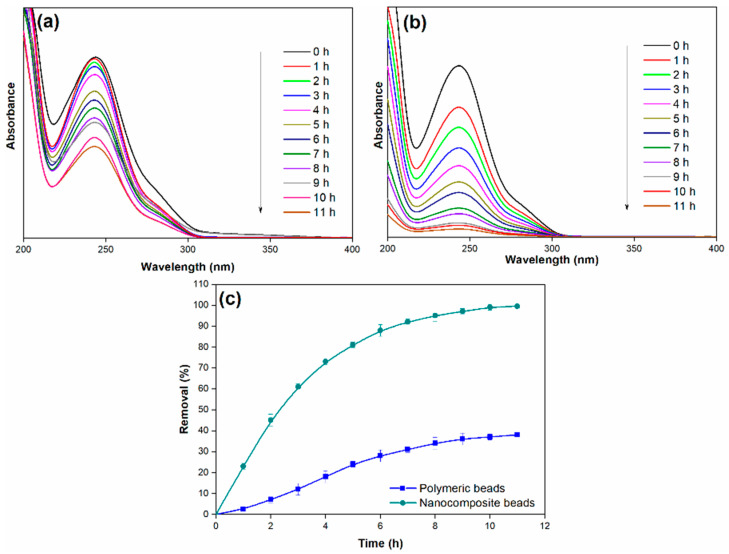
(**a**) Adsorption spectra of paracetamol on polymeric beads at different adsorption times, (**b**) adsorption spectra of paracetamol on nanocomposite beads at different adsorption times, and (**c**) paracetamol removal efficiency over varying adsorption times (adsorbent dosage = 0.3 g, concentration = 100 mg/L, pH = 7, adsorption time = 11 h, solution volume = 25 mL).

In the investigation of the adsorption process, this paper explored the influence of several operational parameters, including the initial paracetamol concentration, adsorption time, solution pH, and adsorbent dosage. The effect of adsorption time, specifically up to 11 h, was studied by introducing a constant amount of nanocomposite beads (0.3 g) into 25 mL of simulated paracetamol-containing wastewater (initial concentration of 100 mg/L) at ambient temperature and pH 7. Figure 8 illustrates that the efficiency of paracetamol removal consistently increased with time, reaching its maximum removal efficiency at the equilibrium point, which occurred at 11 h and resulted in an impressive 99% removal efficiency.

The outstanding removal performance of the nanocomposite beads can be attributed to their unique structural and chemical properties. The interconnected macroscale grooves and enhanced thermal stability observed in the nanocomposite beads contribute to their improved adsorption capacity. Additionally, the higher negative zeta potential exhibited by the nanocomposite beads enhances their interaction with positively charged paracetamol molecules. The presence of oxygen-containing functional groups on the surface, as revealed by XPS and FTIR, likely plays a crucial role in hydrogen bonding and other attractive interactions with paracetamol.

#### 3.2.3. Effect of Paracetamol Concentration

**Figure 9:**
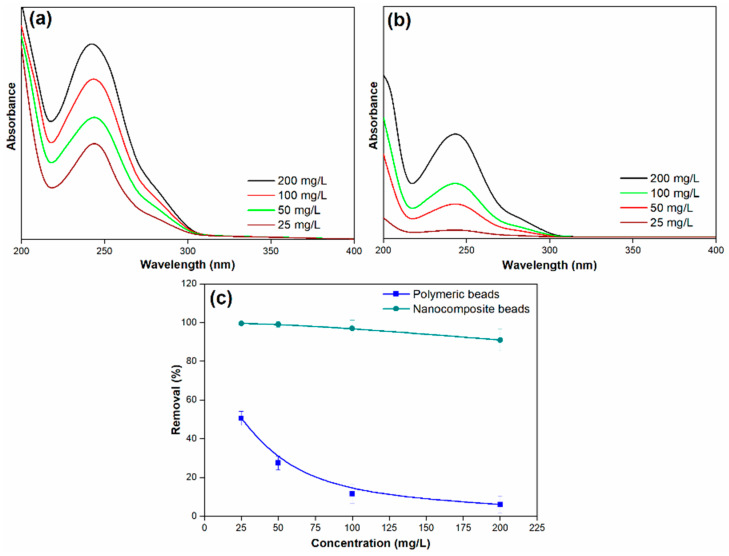
(**a**) Adsorption spectra of paracetamol on polymeric beads at various concentrations, (**b**) adsorption spectra of paracetamol on nanocomposite beads at various concentrations, and (**c**) paracetamol removal efficiency across different concentrations (adsorbent dosage = 0.3 g, concentration = 25 mg/L to 100 mg/L, pH = 7, solution volume = 25 mL).

The impact of the initial paracetamol concentration on the percentage of paracetamol removal by both polymeric and nanocomposite beads is a crucial aspect of understanding the adsorption process and its efficiency in different concentration scenarios. In this study, varying initial concentrations of paracetamol were examined at ambient temperature and a pH of 7, and the results are presented in Figure 9.

For the polymeric beads, it is evident that the removal efficiency significantly decreased as the initial concentration of paracetamol increased. The removal efficiency dropped from 51% to 6% as the initial concentration of paracetamol ranged from 25 to 200 mg/L. This decrease in removal efficiency with rising paracetamol concentration is attributed to the increased number of collisions between paracetamol molecules and the surface of the adsorbent beads. In other words, at higher initial paracetamol concentrations, there is a steeper concentration gradient, creating a stronger driving force for paracetamol molecules to adsorb onto the surface of the beads. This enhanced driving force results in a higher degree of competition for available adsorption sites, causing a reduction in the removal efficiency. Conversely, at lower initial paracetamol concentrations, the paracetamol ions quickly adsorb to the bead surface, leading to a rapid attainment of adsorption equilibrium.

In contrast, the nanocomposite beads exhibited a more robust response to changes in the initial paracetamol concentration. While the removal efficiency did decrease as the initial concentration of paracetamol increased, the decline was marginal. The removal efficiency ranged from 99% to 91% as the initial concentration of paracetamol was varied. This phenomenon can be attributed to the enhanced properties of the nanocomposite beads, which provide a larger number of active surface sites for adsorption. The presence of GO in the nanocomposite beads contributes to a higher number of available binding sites for paracetamol molecules, allowing for more efficient adsorption even at elevated initial concentrations. This characteristic minimizes the effect of increased paracetamol concentration on the removal efficiency compared to the polymeric beads.

#### 3.2.4. Effect of Solution pH

**Figure 10:**
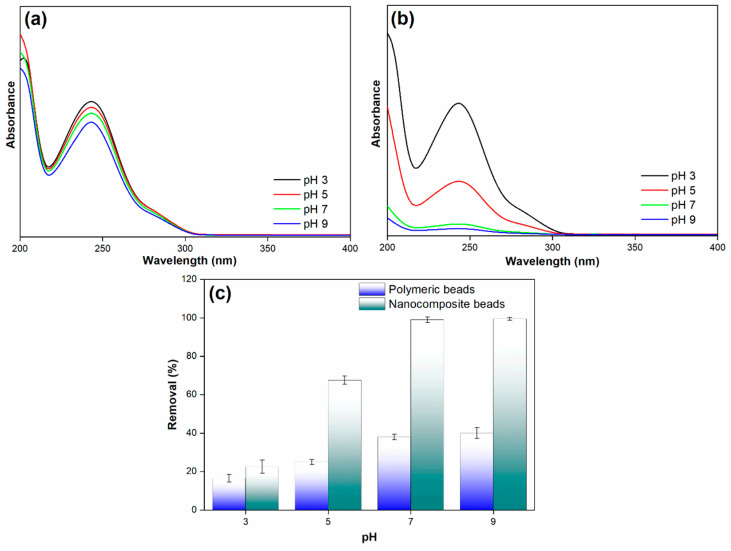
(**a**) Adsorption spectra of paracetamol on polymeric beads at different pH levels, (**b**) adsorption spectra of paracetamol on nanocomposite beads at varying pH levels, and (**c**) paracetamol removal efficiency across diverse pH conditions (adsorbent dosage = 0.3 g, concentration = 100 mg/L, pH = 3 to 9, solution volume = 25 mL).

The pH of the solution is a crucial parameter in the adsorption process, as it plays a pivotal role in influencing the surface charge of adsorbents. The adsorption of a solute is often governed by the protonation and deprotonation of the adsorbate, which, in turn, affects its affinity for the adsorbent. Additionally, the electrostatic attraction between the adsorbent and the adsorbate can be significantly impacted by the solution’s pH, particularly when the attraction of ionic solutes is the predominant adsorption mechanism due to the presence of charged surfaces. In light of these considerations, the study conducted an investigation to understand the influence of pH on paracetamol removal, with a focus on optimizing this parameter for both polymeric and nanocomposite beads. Experimental tests were carried out by varying the pH values within the range of 3 to 9, and the results are depicted in Figure 10.

The findings reveal a significant difference in the paracetamol removal efficiency between the polymeric and nanocomposite beads as the pH changes. Notably, the nanocomposite beads exhibited relatively low paracetamol removal efficiency at a pH of 3. However, as the pH increased, the removal efficiency showed a remarkable improvement, reaching up to 99% at a pH of 7. This high removal efficiency remained constant even at a pH of 9. In stark contrast, the polymeric beads displayed a considerably lower paracetamol removal efficiency under the same pH conditions. The mechanism underlying these observations can be attributed to the interplay between pH and the surface charge of the adsorbents. At a lower pH (pH 3), the surface of the nanocomposite beads may carry a net positive charge, which is less favorable for the adsorption of paracetamol, given its predominantly negative charge [38,39]. As the pH increases toward neutrality (pH 7), the surface charge of the nanocomposite beads becomes more negative. This shift in surface charge enhances the electrostatic attraction between the negatively charged adsorbent and the paracetamol, resulting in increased removal efficiency. At pH 9, the high removal efficiency is maintained as the surface charge remains predominantly negative, promoting effective adsorption.

Conversely, the polymeric beads appear to have a less favorable surface charge configuration for paracetamol removal across the tested pH range. This difference in surface charge properties explains the disparity in removal efficiency between the polymeric and nanocomposite beads at varying pH levels. This result highlights the crucial role of pH in optimizing the adsorption process and the importance of selecting the appropriate adsorbent material with the right surface charge characteristics for specific applications. The research findings provide valuable insights into the interplay between pH and surface charge in adsorption processes, contributing to the development of efficient and tailored adsorption strategies for pollutant removal in diverse environmental remediation applications.

#### 3.2.5. Regeneration of Beads

**Figure 11:**
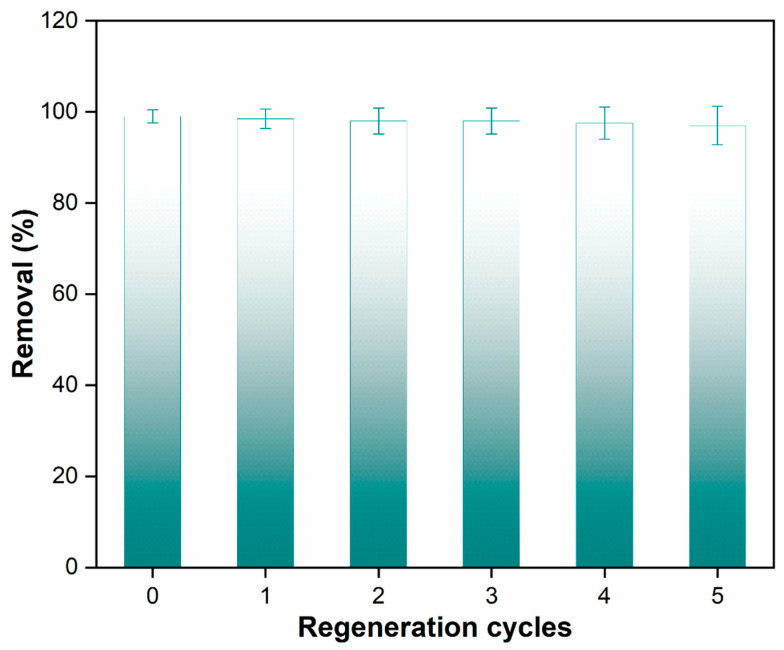
Paracetamol removal efficiency across multiple cycles using nanocomposite beads.

An essential requirement in the adsorption process is the ability of an adsorbent to be regenerated and reused, which has economic and environmental implications. The study addressed this aspect by evaluating the reusability of the nanocomposite beads over multiple cycles. After each adsorption cycle, the spent adsorbent was subjected to a washing process using a mixture of water and ethanol in a 3:1 ratio at a temperature of 50 °C. The results of the regeneration process over five cycles are presented in Figure 11, underscoring the effectiveness of the nanocomposite beads in retaining their adsorption capacity.

The data clearly show that the nanocomposite beads were able to adsorb paracetamol while retaining 97% of their original sorption capacity during the first regeneration cycle. This high retention of adsorption capacity demonstrates the robust reusability of the polymeric nanocomposite beads. This finding holds practical significance as it indicates that these beads can be effectively regenerated using a simple and environmentally friendly washing method. Furthermore, they can be reused multiple times in a batch adsorption system for wastewater treatment. The mechanism underlying the reusability of the nanocomposite beads can be attributed to several factors. The strong interfacial interactions between the GO and the polymeric matrix, as indicated by the characterizations, play a role in stabilizing the structure of the nanocomposite beads even after multiple adsorption–desorption cycles. The well-maintained structural integrity ensures that the beads’ surface characteristics, including the presence of active adsorption sites, are preserved, allowing them to continue efficiently adsorbing paracetamol in subsequent cycles. The paracetamol adsorption capacity of PPSU/GO polymeric nanocomposite beads found in this study is compared with the different adsorbents in Table 2.

## 4. Conclusions

In this investigation, we successfully fabricated PPSU/GO polymeric nanocomposite beads using a one-step phase inversion technique. The primary aim was to address the removal of paracetamol from wastewater. We subjected these beads to comprehensive characterization employing various techniques, including XPS, FTIR, SEM, TGA, and zeta potential analysis. The adsorption performance of the developed adsorbent underwent thorough evaluation with a focus on parameters such as adsorption equilibrium capacity, adsorbent dosage, adsorption time, initial paracetamol concentrations, and solution pH. Our findings highlighted distinct differences in the structural and surface properties as well as adsorption capabilities between the polymeric beads (pure PPSU) and the nanocomposite beads. XPS and FTIR analyses revealed an increased presence of functional groups on the surface of nanocomposite beads following the introduction of GO into the polymer matrix. Additionally, SEM images illustrated a spherical structure with interconnected macroscale grooves in the nanocomposite beads. Furthermore, the zeta potential measurements indicated a substantial enhancement, with the polymeric beads registering −10 mV, while the nanocomposite beads exhibited a significantly improved zeta potential of −34 mV at pH 7. Regarding paracetamol removal efficiency, the polymeric beads exhibited comparatively lower adsorption efficiency, whereas the incorporation of GO into the polymer matrix led to a substantial improvement. The nanocomposite beads demonstrated an exceptional paracetamol removal efficiency of 99% and displayed a notable adsorption capacity. Furthermore, our study showcased the regenerability of the nanocomposite beads through a simple washing method, validating their suitability for multiple adsorption cycles. Even after five successive regeneration and adsorption cycles, the nanocomposite beads retained 97% of their original sorption capacity, emphasizing their practical and sustainable application in batch adsorption systems for wastewater treatment.

## Figures and Tables

**Table 1:** Structure and physicochemical properties of paracetamol.

Drug	Structure	Water Solubility (mg/L)	Molecular Weight (g/mol)	pK_a_	Absorption Wavelength (nm)
Paracetamol	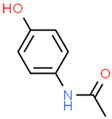	1.40 × 10^4^	151.1	9.5	243

**Table 2:** Comparison of the removal efficiency of paracetamol using different adsorbents.

Adsorbents	Models	pH	Concentration of Feed (mg/L)	Adsorption Capacity (mg/g)	Ref.
Activated carbons and silica gel	Adsorption isotherms and kinetics	3	25	560	[40]
clay-based activated carbon composite	Adsorption isotherms and kinetics	9	50	4.67	[18]
Activated carbon	Adsorption isotherms and kinetics		5	123	[41]
Fe3O4@C matrix	Adsorption isotherms and kinetics	3	10	142.01	[42]
CuONPs	----	7	20	72.46	[43]
PPSU/GO nanocomposite beads	---	7	100	8.25	this study

## Data Availability

Data is contained within the article.
